# Changes in vinegar quality and microbial dynamics during fermentation using a self-designed drum-type bioreactor

**DOI:** 10.3389/fnut.2023.1126562

**Published:** 2023-02-22

**Authors:** Wenxiu Wang, Fan Zhang, Xinpeng Dai, Yaqiong Liu, Jianlou Mu, Jie Wang, Qianyun Ma, Jianfeng Sun

**Affiliations:** ^1^College of Food Science and Technology, Hebei Agricultural University, Baoding, China; ^2^Hebei Technology Innovation Centre of Agricultural Products Processing, Baoding, China; ^3^Sino-US and Sino-Japan Joint Centre of Food Science and Technology, Baoding, China

**Keywords:** microbial community diversity, fermentation, volatile compounds, heatmap package, bioreactor

## Abstract

The bioreactor based on solid-state fermentation technology has been developed for vinegar production, standardization of fermentation process and stabilization of vinegar quality. The microbial community diversity, and volatile compounds of six cultivars of vinegar samples fermented in a self-designed solid-state fermentation bioreactors were investigated using Illumina MiSeq platform and gas chromatography mass spectrometry (GC-MS) technology. The correlations between the richness and diversity of microbiota and volatile profiles, organic acids, as well as physicochemical indicators were explored by R software with the coplot package. The findings indicated that *Acetobacter*, *norank-c-Cyanobacteria*, and *Weissella* played key roles during fermentation process. *Norank-f-Actinopolyporaceae*, *norank-c-Cyanobacteria*, *Pediococcus*, and *Microbacterium* had significant correlations with the physicochemical characteristics. The most common bacterial species were associated with a citric acid content, whereas the least number of bacterial species correlated with malic acid content. Findings could be helpful for the bioreactor optimization, and thus reaching the level of pilot scale and industrialization.

## 1. Introduction

Chinese grain vinegar is a traditional fermented condiment, which plays an indispensable role in an individual’s daily diet (1). Traditional vinegar fermentation has evolved into the classic solid-state fermentation (SSF) style over thousand years (2). In general, vinegar fermentation goes through three steps, including starch saccharification, alcohol fermentation, and acetic acid fermentation (AAF) (3), among which AAF is the most important step as the acetic acid and flavor compounds are formed during the fermentation (4). During the fermentation process, the complex microbial community can provides a variety of enzymes to transform raw materials into a variety of flavor components and functional substances, mainly organic acids and volatile components (5).

In the last decade, the studies on Chinese vinegar research have mainly focused on the dynamics and diversity of microbial communities, and mainly adopted culture-dependent or culture-independent methods (6, 7). Meanwhile, the major microorganisms, volatile flavor compounds, organic acids and amino acids have been identified. Li et al. (8) investigated the bacterial dynamics and metabolite changes in solid-state AAF of Shanxi aged vinegar and discovered eight organic acids, 16 free amino acids, and 66 aroma compounds. Zhou et al. (9) explored the volatile aroma patterns of Beijing rice vinegar in different stages and identified the potential biomarkers for traditional Chinese cereal vinegar. In addition, Ai et al. (10) investigated the microbial diversity of Sichuan bran vinegar, and *Lactobacillus*, *Acetobacter*, *Trichoderma*, and *Candida* were considered as the dominant genera. These studies help us to better understand the mechanism of traditional vinegar fermentation process, as well as the dynamic changes of volatilization, physicochemical properties, and microbial community structure during fermentation.

Currently, Chinese vinegar is generally produced under open and non-sterile environmental conditions (11). These fermentation methods have been used for many years. However, there are still plenty of shortcomings, such as a large occupation area, low degree of mechanization, heavy labor intensity, and low efficiency. These drawbacks make the fermentation process uncontrollable, leading to the inconsistency of quality between different batches (12). Therefore, the traditional fermentation is inadequate to satisfy the practical requirements of modern vinegar production. To address these deficiencies, it is necessary to improve the mechanization and intensive level of SSF of vinegar.

The rapid development of bioreactors based on SSF technology opens a new way for vinegar production, standardizing fermentation process and stabilizing vinegar quality (13). It has the advantages of short fermentation time, controllable working environment and a high degree of mechanization when compared with the traditional fermentation method. In recent years, with the in-depth study of SSF bioreactor, it has been applied in the cultivation of animal and plant cell lines, and production of enzymes (14), ethanol (15), organic acids, pigments (16), and beer.

Combining the principle of multilateral co-fermentation of traditional vinegar SSF with mechanized equipment, the rotary drum type vinegar SSF bioreactor was designed to suit the vinegar fermentation process in our preliminary study. The bioreactor is composed of a power system, baffle, ventilation, and vinegar drenching device, which can complete the steps of inoculation, fermentation, vinegar drenching, and vinegar fumigation in the bioreactor. The baffle device combines the functions of cooling and stirring into one, which reduces the minimum speed of the reactor and reduces energy consumption. The intermittent vinegar drenching process and vinegar fumigation methods were also proposed for the characteristics of the bioreactor. The intermittent rotation strengthens heat and mass transfer, which is beneficial for the growth of microorganisms. In addition, the convective transport is enhanced by increasing the surface contact of the base with wet air and cooling water due to the mixing process. Unfortunately, despite these advantages, the utilization of bioreactors to produce vinegar on an industrial scale has not yet been fully realized. Thus, in order to further improve the design of the bioreactor to meet the needs of industrial scale production, it is essential to deeply understand the fermentation performance of the bioreactor and explore the changes of metabolites during fermentation. In addition, since the coexistence of various microorganisms has a significant influence on the unique flavor and taste of vinegar, a deep understanding of the microbial composition is also required.

Therefore, the aim of this study is to deeper insight into the changes in vinegar quality indexes and microbial dynamics during AAF in this self-designed rotary drum bioreactor. The objectives of this study were to monitor the changes of physicochemical properties, organic acid content and volatile flavor components. The diversity and succession of microbial communities were explored using high-throughput sequencing technology. Furthermore, the potential correlation between dominant bacteria and vinegar quality indexes was determined through multivariate data analysis. The findings in this study could be helpful for the bioreactor optimization, and thus reaching the level of pilot scale and industrialization.

## 2. Materials and methods

### 2.1. Materials and reagents

The fermentation raw materials including flour, bran, rice chaff, and Daqu were collected from the local farmers market of Baoding city in Hebei Province, China. The high-activity yeast culture was purchased from Angel Yeast Co., Ltd. Amylase (1 × 10^5^ U/ml) and glucoamylase (1 × 10^5^ U/ml) were purchased from Shanghai Yuanye Biotechnology Co., Ltd. (Shanghai, China). Lactic acid, acetic acid, oxalic acid, citric acid, malic acid, succinic acid, and tartaric acid were chromatographic grade and purchased from Shanghai Yuanye Biotechnology Co., Ltd. The DNA extraction kit was obtained from United States Omega Bio-Tek (Winooski, VT, United States). Other standard compounds were of analytical purity and purchased from Tianjin Tianli Chemical Reagent Co., Ltd. (Tianjin, China).

### 2.2. Fermentation of vinegar and sample collection

**Figure 1:**
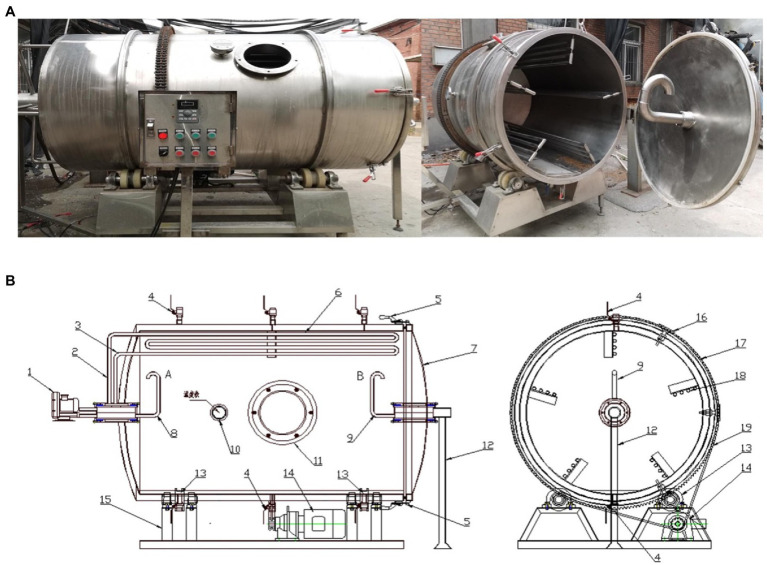
The self-designed drum-type bioreactor: **(A)** Physical map; **(B)** Structural sketch.

In this study, the SSF of vinegar was carried out using a self-designed rotary drum bioreactor, as shown in Figure 1. The motor drives the bioreactor to rotate continuously.

*Composition:* the reactor is composed of a tank, inlet, baffle, support, fan, transmission device, tug, air inlet pipe, air outlet pipe, water inlet pipe, water outlet pipe, observation hole, sampling hole, vinegar spraying sieve plate, and vinegar spraying pipe.

*Fermentation processing:* the feed inlet of the biochemical reactor is upward. First, flour, bran, rice husk, and Daqu were added to the bioreactor at mass ratio of 1:2:1:0.1. The solid material entered the reactor through the material inlet *via* the conveyor belt. The reactor was rotated for 5 min through the gear transmission, and the materials were rotated under the action of the inner baffle of the reactor. Afterwards, the materials automatically fell and were mixed with the rotation. The liquid materials (water, activated yeast, amylase, and glucoamylase) were added into the reactor through the inlet pipe, and were stirred entirely by rotating the bioreactor for 20 min, which promoted vinegar fermentation. At the stage of saccharification and alcohol fermentation, the fermentation culture temperature was maintained at 28–32°C. The oxygen content at the outlet pipe and the internal material temperature were kept at 18–20% and 32–35°C, respectively. On the 19th day of the fermentation, the total acid contents in the vinegar declined, which suggested the end of the AAF stage. Consequently, the fermentation proceeded into the maturation stage, lasting for 5–7 days. After the fermentation, the acetic acid in the fermented grains of solid vinegar was soaked into the water through the water inlet pipe. After the immersion, the vinegar permeated into the juice tank through the solid–liquid isolation sieve plate, and then flowed into the storage tank *via* the bottom vinegar pouring pipe. Afterwards, one side of the tank was opened, the reactor was rotated, and the fermented grains were released immediately. When fumigating vinegar, high-temperature steam was injected into the baffle to heat the vinegar grains.

During fermentation, 100–150 ml of vinegar samples from three vinegar drenching valves were collected on the 1st, 4th, 7th, 11th, 15th, and 19th day of the solid-state acetic acid fermentation process, and then mixed thoroughly to ensure the uniformity and representativeness of the samples. All samples were stored at −80°C until used for further analysis.

### 2.3. Physicochemical properties analysis

The changes of physicochemical properties, including ethanol, total acid, involatile acid compounds, and reducing sugar content of the collected vinegar samples were determined according to GB 5009.225-2016 (National food safety standards, determination of ethanol concentration in wine), GB/T 12456-2008 (Determination of total acid in foods), GB 5009.235-2016 (National food safety standards, determination of amino nitrogen in food), and GB 5009.7-2016 (National food safety standards, determination of reducing sugar in food), respectively.

### 2.4. Organic acids analysis

Organic acids were measured by high-performance liquid Chromatography (HPLC; 1200 Agilent, Santa Clara, CA, United States) referring to a previous method (17). The standard solutions of acetic acid (2.5 mg/ml), lactic acid (1.5 mg/ml), oxalic acid (0.05 mg/ml), tartaric acid (0.1 mg/ml), malic acid (0.05 mg/ml), citric acid (0.75 mg/ml), and succinic acid (0.1 mg/ml) were prepared and filtered into a liquid phase bottle through a 0.22-μm water-soluble filter. Afterwards, 5.0 ml of vinegar sample was treated with 2.0 ml of potassium ferrocyanide (106 g/L) and 2 ml of zinc sulfate (300 g/L), centrifuged at 2,500 *g* for 5 min, and then the supernatant was filtered through a 0.22-μm filter prior to HPLC analysis. The instrument used was equipped with an automatic injector and a photodiode array detector UV at 210 nm. Separation was realized using a Sep-Pak C18 column (5 μm, 4.6 × 250 mm) at 30°C. The mobile phase was 0.02 mol/L NaH_2_PO_4_, with a flow rate of 0.9 ml/min, and the injection volume of samples was 10 μl. Finally, each organic acid was quantified by calculating the peak area.

### 2.5. Volatile profiles analysis

Volatile compounds of the vinegar samples were analyzed by utilizing head space solid phase microextraction coupled with gas chromatography mass spectrometry (HS-SPME-GC/MS) analysis (7890A-5975C, Agilent, Santa Clara, CA, United States) (18). Briefly, 6 ml of vinegar and 1.5 g of NaCl were pipetted into a 15 ml of headspace vial, tightly capped and equilibrated at 40°C in a thermostatic bath. The sample was then extracted by SPME head at the same temperature for 40 min. After extraction, the fiber was immediately inserted into the injection port of GC-MS to thermally desorb the analyte at 240°C for 6 min.

GC/MS analyses were performed on an Agilent 7890A GC and an Agilent 5953C MS equipped with a capillary column (30 m × 0.25 mm, 0.25-μm thickness, TG-WAXMS, Thermo Scientific) (19). The GC operation condition was as follow: inlet temperature of 250°C, a split ratio of 1:1, helium carrier gas flow of 1 ml/min. The oven temperature was maintained at 40°C for 5 min, followed by an increase of 10°C/min to 180°C, and then programmed to 230°C at 5°C/min, and held for 10 min. The MS was generated in the electron impact mode at 70 eV of ionization energy using the full scan mode (14–400 amu). The temperature of MS source and quadrupole was set at 230 and 150°C, respectively.

The volatile compounds were then identified by matching the MS with the NIST05 mass spectral database and quantitatively calculated using the peak-area normalization method. All samples were analyzed in triplicate.

### 2.6. Microbial community analysis

#### 2.6.1. DNA extraction and qPCR analysis

To monitor the dynamic changes in a microbial community, samples were collected periodically on the surface of fermentation materials in the bioreactor on the day 1, 4, 7, 11, 15, and 19 during the AAF process. For each day, each 150 g of sample was collected at three different locations and mixed thoroughly to ensure the uniformity and representativeness of samples. All samples were put into sterile sealed bags and stored in a freezer at −80°C. Then DNA extraction was performed using the Soil DNA Kit (Omega Bio-Tek, Norcross, GA, United States). The concentration of extracted DNA was measured by a NanoDrop 2000 UV-vis spectrophotometer (Thermo Scientific, Wilmington, MA, United States) and checked by 1% agarose gel electrophoresis. Bacterial primers 338F (5′-ACTCCTACGGGAGGCAGCAG-3′) and 806R (5′-GGA CTACHVGGGTWTCTAAT-3′) with specific barcode were employed to amplify the V3-V4 region of bacterial 16S rRNA genes by thermocycler PCR system (ABI GeneAmp® 9,700, Waltham, MA, United States). The PCR reaction was run as the method from our lab, which has been published by Ma et al. (20).

#### 2.6.2. Sequencing and data analysis

For the sequencing and data analysis, the method described by Ma et al. (20) was followed. Trimmomatic quality-filtered raw fastq files before FLASH combined them with the following standard. The reads were truncated at any site with an average quality score of 20 over a 50 bp sliding window; sequences with overlap longer than 10 bp were merged according to their overlap with a mismatch of no more than 2 bp; and sequences of each sample were separated according to barcodes (exactly matching) and Primers (two nucleotides mismatched were allowed). Reads with unclear bases were filtered out. UPARSE (version 7.1)^1^ was used to cluster operational taxonomic units (OTUs) using a 97 percent similarity cut-off. RDP Classifier (version 2.11)^2^ was used to compare the taxonomy of each 16S rRNA gene sequence to the SILVA (version 132)^3^ 16S rRNA database. The confidence level was set at 70%.

### 2.7. Data analysis

All the determinations were conducted in triplicate unless otherwise stated. Statistical analysis was performed using SPSS 22.0 (SPSS Inc., Chicago, IL, United States) and Origin9.1. Principal component analysis (PCA) was performed using the i-sanger tools to cluster samples according to the relative abundance of microbes. The correlation between bacterial community and physicochemical indicators, organic acids and volatile compounds were investigated by Spearman’s correlation coefficient at |*ρ*| > 0.7 with statistically significance (*p* < 0.01). The results were visualized with heatmap by using R software with the “corrplot” package.

## 3. Results and discussion

### 3.1. Physicochemical properties and organic acids contents during fermentation

**Figure 2:**
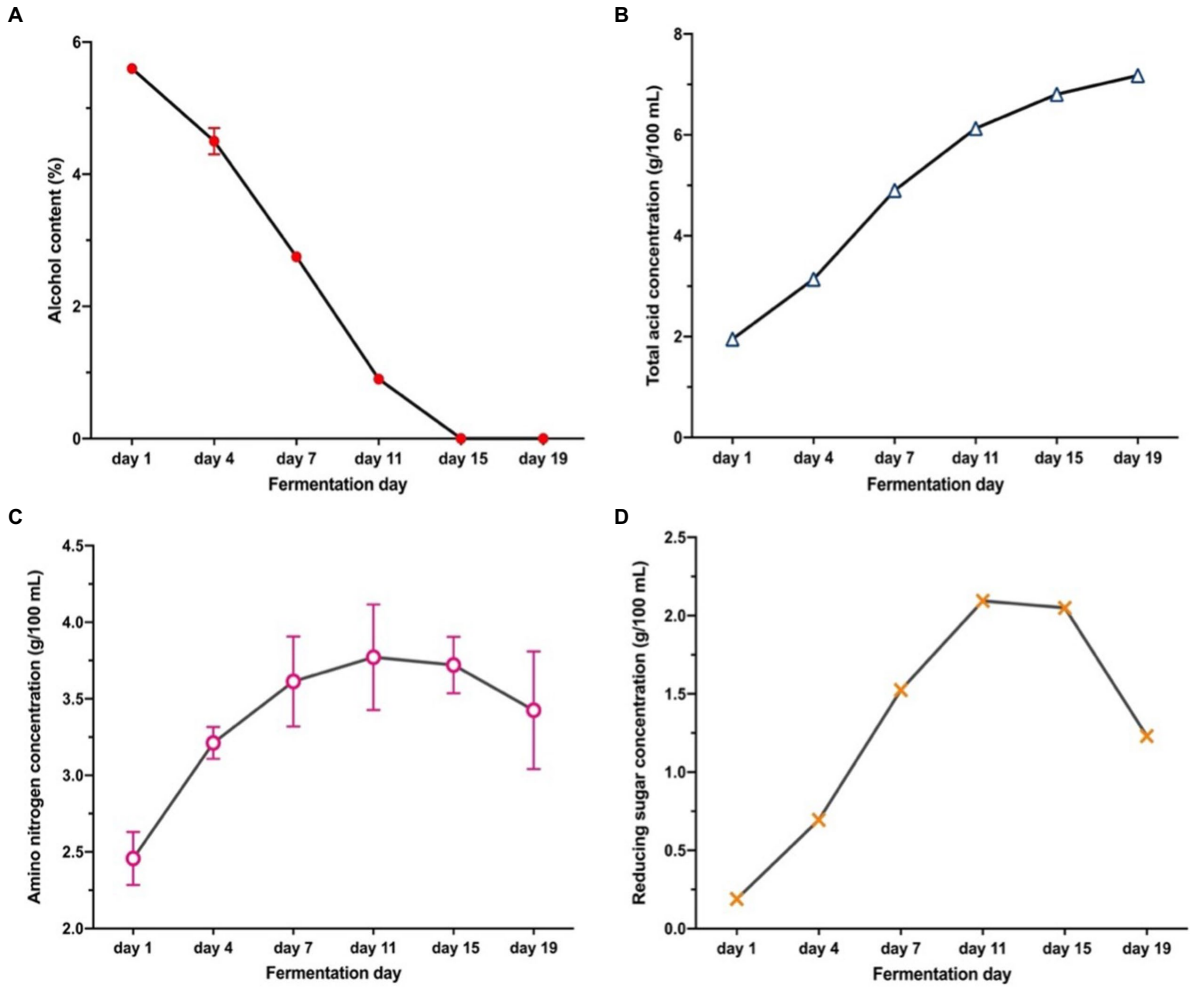
Changes in physical and chemical indicators during vinegar fermentation: **(A)** Ethanol content; **(B)** Total acid; **(C)** Amino nitrogen; **(D)** Reducing sugar content.

The changes in physicochemical indices including the ethanol (**2A**), total acid (**2B**), amino nitrogen (**2C**), and reducing sugar contents (**2D**) are shown in Figure 2. These characteristics are the key indicators of process control, and the factors of forming unique flavor. The highest ethanol content of 5.6% was observed at the initial stage of AAF, whereas it dramatically decreased to 0% on the 15th day of AAF. During AAF, ethanol is oxidized to acetaldehyde under the catalysis of ethanol dehydrogenase, and then acetaldehyde is oxidized to acetic acid under the catalysis of aldehyde dehydrogenase (21). Thus, it can be seen a significant increase in the total acid content (Figure 2B) from the 1st (1.95 g/100 ml) day to the 19^th^ day (7.18 g/100 ml) of AAF. This observation was in agreement with previous studies (21). Amino nitrogen is related to the degradation of nitrogen-containing compounds (22). As shown in Figure 2C, amino nitrogen concentration showed an increased tendency from 2.46 to 3.45 g/100 ml. According to the Figure 2D, the reducing sugar concentration reached a maximum of 2.10 g/100 ml on the 11th fermentation day, and then decreased to 1.23 g/100 ml at the end of the AAF process. The microbial communities and metabolites are involved in the complicated interactions during AAF, leading to the degradation of oligosaccharides and polysaccharides into the reducing sugar (2). At the later fermentation stage, the decrease in reducing sugar content was due to the growth of microorganisms could be at the expense of reducing sugar (23).

**Figure 3:**
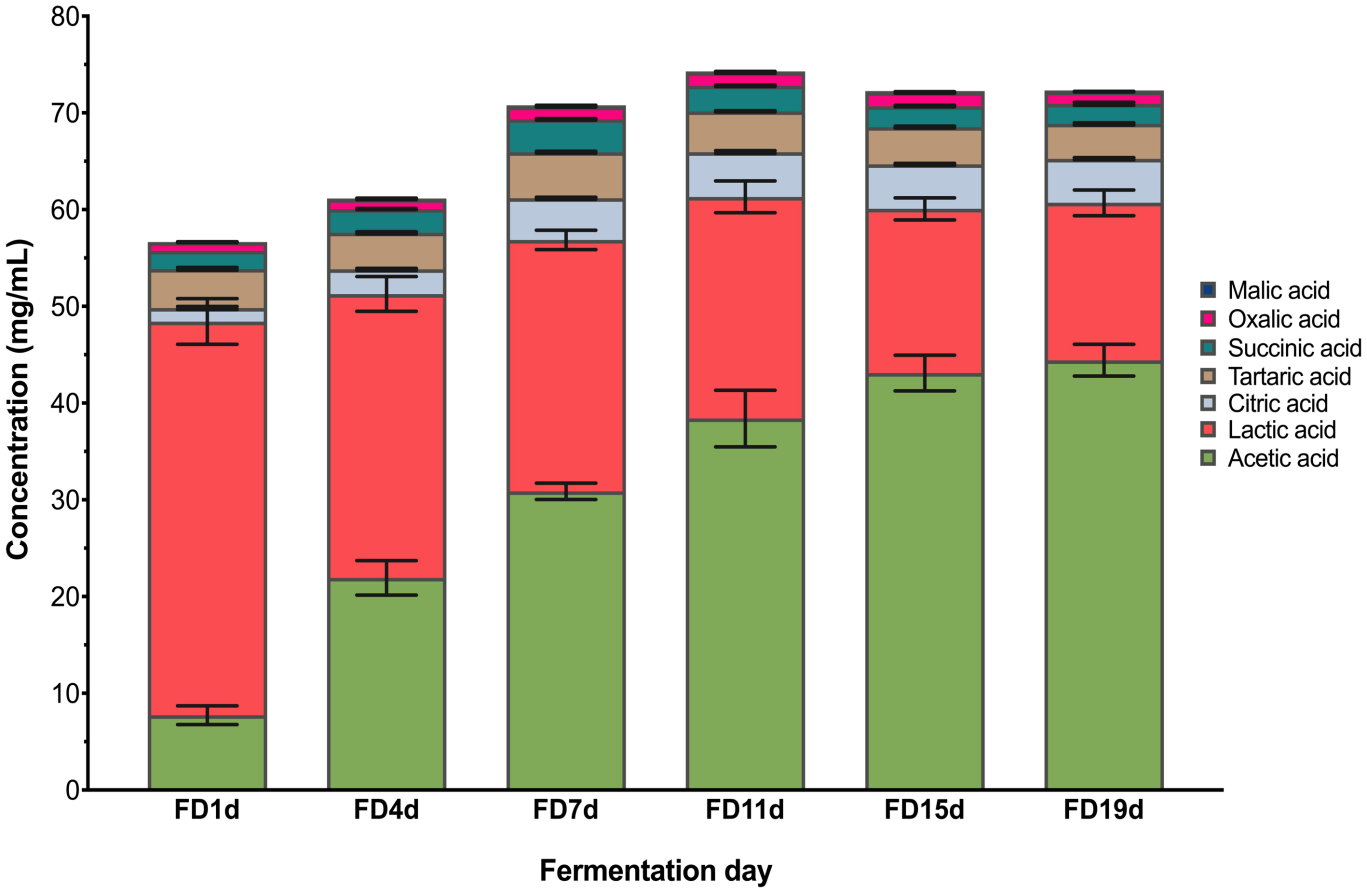
Changes in organic acids during vinegar fermentation.

Dynamics of organic acid contents during AAF are shown in Figure 3. Seven kinds of organic acids including acetic acid, lactic acid, oxalic acid, succinic acid, tartaric acid, citric acid, and malic acid were detected by HPLC analysis. Acetic acid and lactic acid are the two dominant organic acids observed in this study, taking accounts 83.95% of the total organic acids at the end of AAF. These acids have been reported as the dominant organic acids in typical Chinese vinegar, such as Shanxi aged vinegar, Zhenjiang aromatic vinegar, and Tianjin duliu vinegar (10). The acetic acid content was sharply increased from 7.74 (1st fermentation day) to 44.44 g/100 ml (19th fermentation day), while the lactic acid content showed a decrease from 40.70 (1st fermentation day) to 16.27 g/100 ml (19th fermentation day). From the 4th day of AAF, the lactic acid content began to decrease. The increased oxygen content made the metabolisms of lactic acid bacteria slow. Simultaneously, the lactic acid was utilized by part of acetic acid bacteria such as acetic anhydride utilized one carbon source, converting the lactic acid into the acetic acid.

The ratio of lactic acid to acetic acid gradually decreasing, and this ratio was less than 1 on the 11th day of fermentation, indicating that the acetic acid content exceeded lactic acid content. During the alcoholic fermentation of the early stage of AAF, the anaerobic condition is favorable for the metabolisms of lactic acid bacteria to produce lactic acid. On the 3rd day of fermentation, the acetic acid bacteria were introduced from the outside and began to proliferate in the reactor, leading to the rapid production of the acetic acid. Consequently, the content of acetic acid exceeded the lactic acid content, accounting for the largest proportion of total organic acids. However, the high lactic acid content can soothe the pungent taste of acetic acid (24). Additionally, the contents of citric acid and tartaric acid were also abundant during AAF, increasing from 1.40 to 4.56 g/100 ml and decreased from 4.04 to 3.62 g/100 ml during AAF, respectively (*p* < 0.05). The succinic acid content showed a gradual upward trend, while the malic acid content decreased during AAF.

### 3.2. Analysis of volatile compounds during fermentation

Volatile compounds give vinegar a special flavor. Most volatile compounds are derived from microbial metabolic reactions, while others can be derived from degradation or Maillard reactions (25). In this study, 64 volatile components were identified and quantified during AAF, as shown in Table 1. Based on their retention time, they were classified into esters, alcohols, acids, phenols, aldehydes, ketones, and heterocycles. At the early stage of fermentation, the concentration of all volatile compounds was low (39.94 mg/100 ml). After the yeast culture, fermentation gradually entered the alcoholic fermentation stage, aroma concentration bursting. The total concentration of these compounds reached 1203.91 mg/100 ml at the end of AAF.

**Table 1:** Identification of different compounds during the fermentation processing by using rotary drum reactor.

Variables	Compounds	Relative content (%)					
		F1d	F4d	F7d	F11d	F15d	F19d
Esters	Ethyl acetate (S1)	7.97 ± 1.21	10.10 ± 1.20	13.68 ± 2.45	16.27 ± 2.67	8.04 ± 1.13	3.25 ± 0.24
	Ethyl caproate (S2)	1.19 ± 0.02	3.53 ± 0.56	3.39 ± 0.47	2.28 ± 0.43	1.75 ± 0.15	1.30 ± 0.01
	Ethyl caprylate (S3)	4.72 ± 0.23	4.59 ± 0.83	3.25 ± 0.63	2.51 ± 0.27	1.09 ± 0.06	ND
	Ethyl nonyl (S4)	0.82 ± 0.04	0.55 ± 0.02	0.39 ± 0.03	0.01 ± 0.00	ND	ND
	Ethyl decate (S5)	0.62 ± 0.01	1.27 ± 0.02	0.45 ± 0.01	0.52 ± 0.01	0.51 ± 0.01	ND
	Diethyl succinate (S6)	1.39 ± 0.01	0.88 ± 0.03	2.43 ± 0.02	1.67 ± 002	0.98 ± 0.03	ND
	Phenethyl acetate (S7)	1.20 ± 0.02	2.38 ± 0.43	3.87 ± 0.03	3.74 ± 0.13	4.56 ± 1.32	4.68 ± 0.86
	Benzyl acetate (S8)	ND	ND	0.25 ± 0.01	ND	ND	ND
	Ethyl myristate (S9)	0.98 ± 0.03	0.73 ± 0.04	0.28 ± 0.00	0.32 ± 0.07	ND	ND
	Propionolactone (S10)	3.88 ± 0.21	3.44 ± 0.02	4.68 ± 0.12	4.30 ± 0.18	4.25 ± 1.29	2.62 ± 0.48
	Propionolactone (S11)	ND	0.03 ± 0.00	0.26 ± 0.00	0.30 ± 0.00	0.57 ± 0.05	ND
	Ethyl palmitate (S12)	6.94 ± 0.78	4.37 ± 0.58	3.82 ± 0.26	3.04 ± 0.69	4.07 ± 0.08	ND
	Ethyl laurate (S13)	0.93 ± 0.05	0.45 ± 0.00	0.53 ± 0.01	0.58 ± 0.10	0.64 ± 0.01	ND
	Ethyl oleate (S14)	1.12 ± 0.02	0.54 ± 0.01	0.83 ± 0.02	0.87 ± 0.08	ND	ND
	Ethyl linoleate (S15)	0.93 ± 0.01	0.97 ± 0.02	2.21 ± 0.23	0.98 ± 0.01	ND	ND
	Isoamyl acetate (S16)	0.71 ± 0.01	0.41 ± 0.01	4.89 ± 1.29	1.62 ± 0.00	1.62 ± 0.02	2.79 ±
	Ethyl heptanate (S17)	0.82 ± 0.00	0.53 ± 0.02	0.41 ± 0.01	0.22 ± 0.00	0.47 ± 0.00	ND
	Hexyl acetate (S18)	1.88 ± 0.19	1.81 ± 0.03	5.12 ± 1.29	0.93 ± 0.02	0.54 ± 0.02	0.34 ± 0.02
	Heptane acetate (S19)	ND	ND	0.17 ± 0.02	0.20 ± 0.00	0.44 ± 0.00	ND
	Ethyl benzoate (S20)	0.84 ± 0.06	0.66 ± 0.02	0.66 ± 0.04	0.58 ± 0.01	ND	1.12 ± 0.05
	Ethyl phenylacetate (S21)	0.55 ± 0.00	0.25 ± 0.00	0.73 ± 0.02	0.56 ± 0.04	0.85 ± 0.04	ND
	Ethyl 3-phenylpropionate (S22)	0.65 ± 0.01	0.78 ± 0.18	ND	ND	0.78 ± 0.02	ND
	Diethyl azelaite (S23)	2.53 ± 0.04	0.67 ± 0.02	0.31 ± 0.00	0.76 ± 0.03	ND	ND
	Diethyl succinate (S24)	0.66 ± 0.01	0.42 ± 0.01	ND	0.57 ± 0.01	0.96 ± 0.02	ND
	Diethyl succinate (S25)	0.51 ± 0.00	0.71 ± 0.03	0.11 ± 0.00	0.06 ± 0.00	0.98 ± 0.01	ND
Alcohols	Ethanol (S26)	22.44 ± 2.78	11.48 ± 2.46	6.45 ± 1.28	5.07 ± 1.29	3.51 ± 0.78	2.01 ± 0.78
	Isoamyl alcohol (S27)	1.82 ± 0.03	1.50 ± 0.01	1.46 ± 0.39	0.80 ± 0.02	2.12 ± 0.18	2.31 ± 0.49
	Phenyl ethanol (S28)	1.16 ± 0.01	2.06 ± 0.02	3.42 ± 0.89	3.11 ± 0.78	3.73 ± 0.27	3.70 ± 1.29
	Hexanol (S29)	3.91 ± 1.10	3.64 ± 0.76	3.39 ± 1.29	2.05 ± 0.37	1.07 ± 0.21	ND
	Octanol (S30)	1.11 ± 0.00	1.21 ± 0.03	0.16 ± 0.03	ND	ND	ND
	1-Nonanol (S31)	1.05 ± 0.01	0.96 ± 0.24	0.54 ± 0.02	0.32 ± 0.02	0.52 ± 0.04	ND
	Heptanol (S32)	0.87 ± 0.03	0.74 ± 0.28	0.05 ± 0.00	ND	ND	ND
	Isobutanol (S33)	ND	ND	ND	0.05 ± 0.00	ND	ND
	(R) - (-) - 2-butanol (S34)	0.71 ± 0.02	ND	ND	ND	ND	ND
	2,3-butanediol (S35)	3.34 ± 0.02	6.62 ± 1.28	4.26 ± 1.38	1.18 ± 0.02	1.02 ± 0.04	0.33 ± 0.00
	(2R, 3R) - (-) - 2,3-butanediol (S36)	ND	0.55 ± 0.05	0.09 ± 0.01	1.61 ± 0.02	1.12 ± 0.05	0.47 ± 0.01
	Cis-4-decene-1-ol (S37)	1.00 ± 0.04	1.66 ± 0.42	0.02 ± 0.00	0.36 ± 0.01	0.56 ± 0.02	ND
Acids	Acetic acid (S38)	8.21 ± 1.13	8.64 ± 1.39	12.02 ± 2.37	18.14 ± 2.74	18.79 ± 3.78	48.50 ± 9.76
	Caproic acid (S39)	5.49 ± 0.98	6.53 ± 1.18	5.80 ± 1.19	6.80 ± 2.38	11.99 ±	6.68 ± 2.04
	Butyrate (S40)	ND	ND	ND	ND	2.53 ± 0.89	3.73 ± 1.20
	Heptanic acid (S41)	ND	ND	0.36 ± 0.04	ND	2.68 ± 0.48	1.27 ± 0.67
	Bitter (S42)	2.37 ± 0.45	6.76 ± 0.02	1.68 ± 0.02	4.50 ± 0.46	4.68 ± 1.38	2.34 ± 0.45
	Palmitic acid (S43)	ND	ND	0.76 ± 0.04	7.55 ± 1.29	1.29 ± 0.02	1.27 ± 0.38
Phenols	4-vinyl-2-methoxyphenol(S44)	0.42 ± 0.02	0.80 ± 0.00	0.37 ± 0.01	0.27 ± 0.01	0.61 ± 0.06	0.60 ± 0.01
	2-methoxy-4-methylphenol (S45)	ND	ND	0.42 ± 0.02	0.71 ± 0.07	3.24 ± 1.23	2.15 ± 0.55
	4-ethyl-2-methoxyphenol (S46)	0.71 ± 0.04	0.32 ± 0.00	0.37 ± 0.01	0.44 ± 0.01	1.27 ± 0.06	0.79 ± 0.18
	Phenol (S47)	1.09 ± 0.02	0.96 ± 0.01	0.55 ± 0.00	0.40 ± 0.05	0.57 ± 0.02	0.80 ± 0.02
	Guaiacol (S48)	1.09 ± 0.01	1.81 ± 0.01	3.89 ± 0.57	4.12 ± 0.28	4.67 ± 1.29	4.53 ± 1.28
Aldehydes	Benzaldehyde (S49)	0.37 ± 0.02	3.24 ± 0.01	0.92 ± 0.01	1.52 ± 0.43	1.74 ± 0.23	1.31 ± 0.09
	Phenylacetaldehyde (S50)	0.18 ± 0.00	ND	ND	ND	0.85 ± 0.04	1.64 ± 0.58
	2-hydroxy-3-methylbenzaldehyde (S51)	ND	ND	ND	ND	ND	0.15 ± 0.04
	2-hydroxy-6-methylbenzaldehyde (S52)	0.54 ± 0.01	0.41 ± 0.00	2.12 ± 0.03	1.88 ± 0.28	1.36 ± 0.28	1.43 ± 0.05
	Furfural (S53)	ND	ND	ND	ND	ND	ND
Ketone	2-pyrrolidone (S54)	ND	ND	ND	ND	ND	1.74 ± 0.27
	3-hydroxy-2-butanone (S55)	ND	ND	0.13 ± 0.01	1.41 ± 0.24	4.89 ± 1.29	1.77 ± 0.43
	6-methyl-3,5-pentadiene-2-one (S56)	0.37 ± 0.02	1.46 ± 0.34	0.23 ± 0.00	0.43 ± 0.19	0.34 ± 0.02	0.46 ± 0.01
	3-acetyl-2-butanone (S57)	ND	ND	0.08	2.77 ± 0.56	ND	1.87 ± 0.76
	2- piperazine(S58)	ND	ND	ND	ND	1.41 ± 0.13	ND
	2,3-butanedione (S59)	ND	ND	0.17 ± 0.00	ND	ND	ND
Heterocycles	Trichloromethane (S60)	ND	0.31 ± 0.02	0.27 ± 0.04	0.31 ± 0.06	ND	ND
	Sixteen alkane (S61)	ND	ND	ND	ND	0.25 ± 0.00	0.41 ± 0.01
	2,3,5,6-tetramethylpyrazine (S62)	ND	ND	ND	0.92 ± 0.03	1.08 ± 0.03	1.45 ± 0.13
	2,3,5-trimethylpyrazine (S63)	ND	ND	0.25 ± 0.01	ND	0.41 ± 0.01	ND
	3-methyl-bicyclo [4.1.0] heptane (S64)	ND	ND	ND	ND	1.02 ± 0.07	0.41 ± 0.05

Values are mean ± standard deviation (SD), *n* = 3. FD, Fermentation day.

Esters are the most common aroma category in vinegar and contributed to the fruity and baking flavors of the product (26). Herein, 25 esters was recognized in the vinegar, which were produced by microorganisms during AAF or synthesized by acid esterification in the presence of ethanol. The number of ester compounds was increased to 23 on the 7th day of AAF, and then reduced to 6 until the end of AAF. The ethyl acetate content on the 15th day was significantly higher than that of other esters, accounting for 42.03% of the total ester contents.

Alcohols mainly originated from the alcohol fermentation stage that provided the precursors for the synthesis of organic acids (4). In this study, 12 alcohols were identified. Most alcohols showed a decreased tendency during AAF. Ethanol accounts for the highest proportion of all the types of alcohol, decreasing from 22.44 to 3.51% throughout the fermentation process. Octanol and Heptanol were not detected in the later stage.

Acidic compounds have a crucial influence on the sensory characteristics of vinegar (27). A total of six acids were detected with a concentration of 63.79% at the end of fermentation, among which the most abundant was acetic acid, accounting for 80.05% of all the acids contents by the end of fermentation. The sufficient acid content might restrict the growth of other bacteria, and increase the mellow and aftertaste of the fermented products, and improve their flavor. Meanwhile, they can aid in the creation of esters. Aldehydes, phenols, ketones, and pyrazines were also present in minute levels during the experiment.

### 3.3. Dynamics of microbial community during fermentation

After filtering low-quality reads, removing adapters, barcodes, and primers, and detecting chimera, roughly 39,705 to 59,287 effective tags, with various phylogenetic OTUs ranging from 16 to 48 *via* 97 percent sequence identity cutoff, were obtained for bacterial dynamics and diversity. At the genus level, taxonomic affiliations of 97 percent sequence similarity clusters revealed a substantial taxonomic shift during the AAF process.

**Figure 4:**
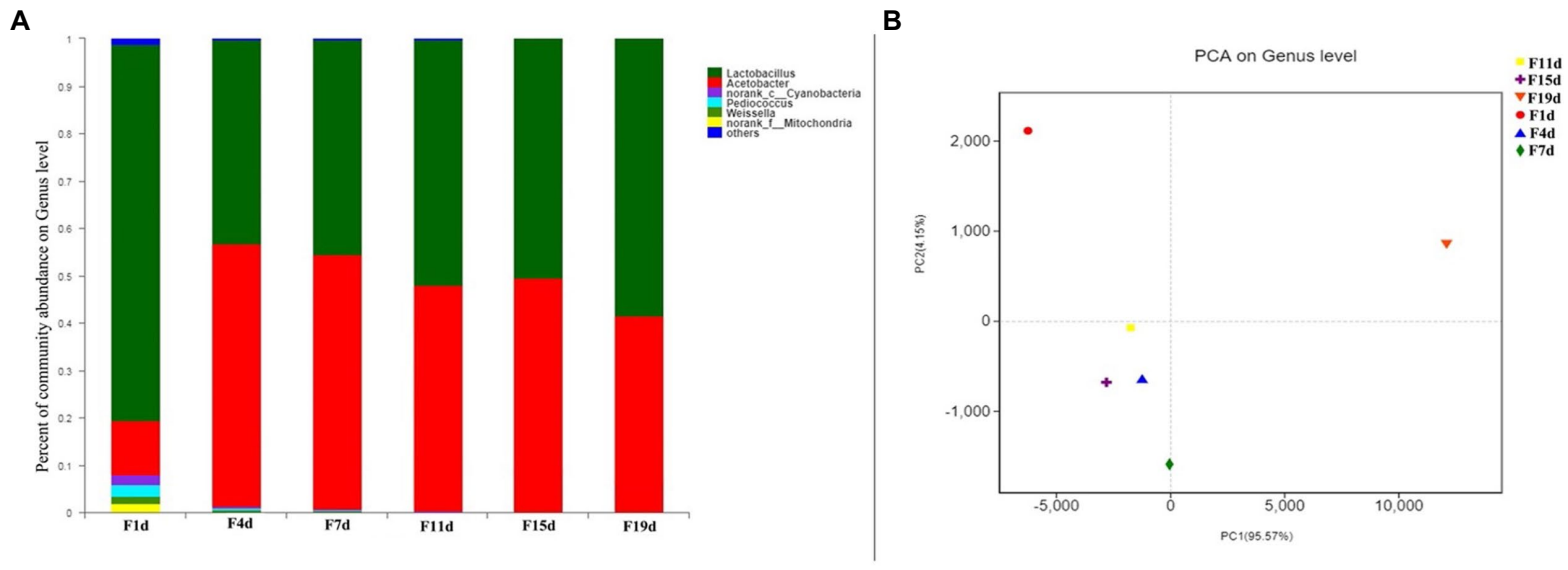
**(A)** Distribution of bacterial community at genus level during vinegar acid fermentation; **(B)** Principal component analysis of microorganism during fermentation.

The bacterial community consisted of 48 genera, including six genera with relative content greater than 1%, which comprised *Lactobacillus*, *Acetobacter*, *Cyanobacteria*, *Pediococcus*, *Weissella*, and *Mitochondria* (Figure 4A). *Lactobacillus* and *Acetobacter* were the most common genera, accounting for more than 90% of all sequences. On days 1 to 19 of AAF, *Lactobacillus* accounted for the greatest percentage, ranging from 43.98 to 78.97 percent. *Lactobacillus* is an anaerobe that thrives during aerobic fermentation but is inhibited by low pH and high acidity. Increased acetic acid levels in fermentation cultures resulted in acidic stress, which favored acid-tolerant bacteria (28). The relative content of *Acetobacter* was only 13.1% on the first day of AAF and increased rapidly to 56.3% on day 4 during fermentation, then decreased slightly, and stabilized between 45 and 50%. *Acetobacter* has effectively dominated the entire fermentation process, and its relative abundance has dramatically increased. After the high acidity and low pH incubation stage, the structure of the bacterial community may be modified to the features of acidophilic and aerobic communities, according to these findings. During the AAF process, changes in modest proportions in the *Cyanobacteria* (4.34–0.04%), *Pediococcus* (1.22–0%), and *Weissella* (0.09–0.01%) groups were also noted. These findings revealed that Lactobacillus and Acetobacter were competing spontaneously. Acetobacter was a fierce competitor in the bacterial community’s succession. Alpha diversity indexes representing the number of OTUs, Sobs, Chao, Shannon, Simpson, Ace, and Coverage index were determined to further confirm whether our sequencing results were sufficient to analyze the food fermentation ecosystem, and these indexes demonstrated that the richness of diversity varied during AAF (Table 2). The Sobs and Ace indicate the actually observed richness value, whereas the Chao is used to estimate OTU counts in samples. The microbiological diversity in samples is depicted by both Simpson and Shannon. Herein, the OTUs, Sobs, Chao, Shannon, and Ace index decreased during the AAF process, while the Simpson index increased significantly. This result illustrates that the solid acid fermentation improves the intestinal flora richness and diversity in vinegar samples. The diversity and richness of gut flora may play a role in human obesity and other chronic disorders. Each sample library’s coverage is represented by coverage. In this investigation, all of the coverage values found are greater than 0.99. This conclusion shows that the sequence in the sample was extremely likely to be discovered, and that the distribution of the bacterial community of samples might be represented by this finding. In addition, the number of microbial genera detected in this study was less than that in traditional fermented vinegar, while the relative abundance of *Lactobacillus* and *Acetobacter* was higher. The reason could be that the drum-type bioreactor is a relatively closed environment and the whole fermentation process is carried out in it, reducing the contact with the external condition, thus reducing the contamination by miscellaneous bacteria, and consequently providing a favorable fermentation environment for microorganisms.

**Table 2:** Richness and diversity indexes from samples at different fermentation stages.

Fermentation day	OTUs	Sobs	Chao	Shannon	Simpson	Ace	Coverage
F-1d	48.00 ± 2.65^a^	48.33 ± 2.52^a^	50.06 ± 3.02^a^	1.08 ± 0.18^a^	0.59 ± 0.07^c^	47.35 ± 4.84^a^	0.9973 ± 0.0002^b^
F-4d	34.33 ± 13.58^a^	35.33 ± 5.51^bc^	41.28 ± 5.71^b^	0.77 ± 0.12^b^	0.65 ± 0.08^bc^	44.28 ± 7.01^a^	0.9964 ± 0.0003^d^
F-7d	33.00 ± 1.00^ab^	37.33 ± 4.16^b^	43.28 ± 2.75^b^	0.63 ± 0.01^c^	0.73 ± 0.01^b^	44.85 ± 4.06^a^	0.9974 ± 0.0002^b^
F-11d	27.33 ± 1.53^c^	30.00 ± 0.00^c^	40.21 ± 10.24^bc^	0.50 ± 0.06^d^	0.78 ± 0.04^ab^	40.89 ± 6.34^a^	0.9964 ± 0.0002^d^
F-15d	19.00 ± 1.00^d^	21.33 ± 3.22^d^	32.42 ± 14.22^bc^	0.46 ± 0.12^ed^	0.78 ± 0.08^ab^	34.76 ± 18.00^a^	0.9982 ± 0.0003^a^
F-19d	16.67 ± 0.58^e^	23.33 ± 2.31^d^	29.83 ± 5.58^c^	0.42 ± 0.02^e^	0.81 ± 0.02^a^	42.03 ± 15.24^a^	0.9973 ± 0.0002^bc^

Values are mean ± standard deviation (mean ± SD, *n* = 3). Values with different letters within the same column differ from each other statistically. F, Fermentation.

To examine the differences and similarities in bacterial Illumina MiSeq sequencing at different fermentation stages, PCA analysis (Figure 4B) was used. Similarity analysis between groups was done on samples at the Genus level, as seen in the figure. In the same group, the obtained values are fairly consistent. The first principal component (PC1) accounted for 91.29% of the total variance, while the PC2 explained a further 8.25%. Based on the bacterial structure, the AAF process was divided into three stages: pre-fermentation stage (1 day), medium fermentation stage (4 days), and late fermentation stage (7–19 days). This division provided a succession of bacterial profiles at different stages of AAF. The OTUs from day 1 lie on the first quadrant, which was characterized mainly by *Lactobacillus*, followed *Acetobacter*, *Cyanobacteria, Pediococcus, Weissella*, and *Mitochondria*. In addition, sample on the 4th day exhibited distinctive characters, showing a significant difference with the bacterial structure of day 1. The patterns of OTUs from days 7, 11, 15, and 19 were well clustered, which were mainly characterized *by Lactobacillus* and *Acetobacter*. These results were in agreement with the microbial community diversity and richness analysis, which could be attributed to the changes in temperature, pH, and acidity during the AAF process.

### 3.4. Correlation analysis between bacterial community and various indicators

**Figure 5:**
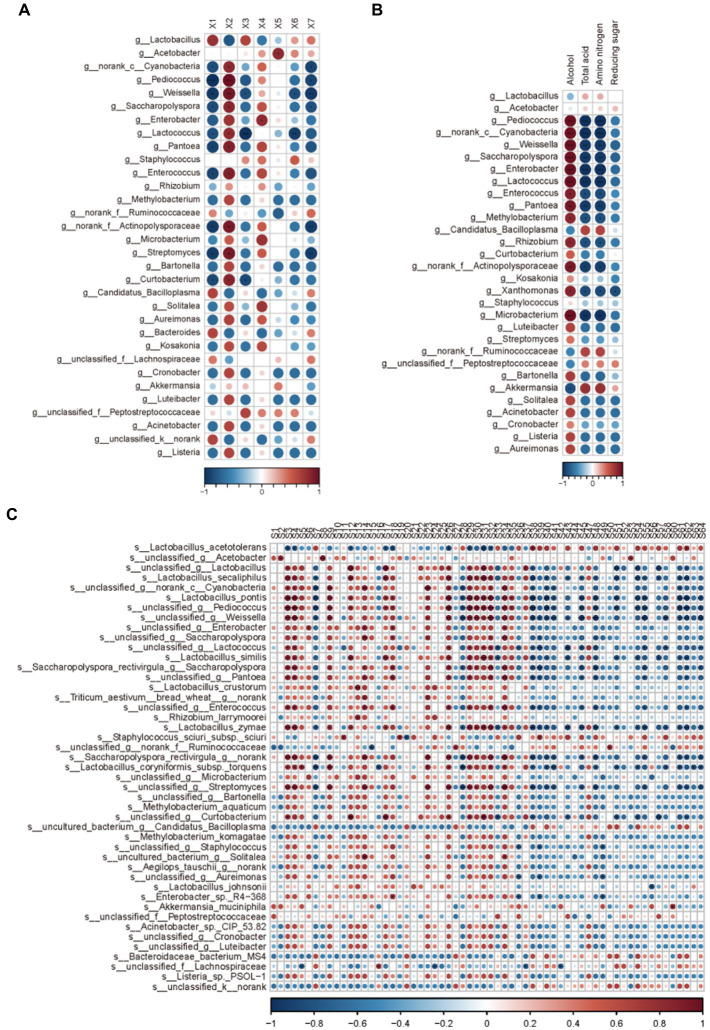
Correlation analysis between microbial community and various indicators: **(A)** organic acids; **(B)** Physical and chemical indicators; **(C)** Volatile compounds.

During the AAF process, microbial diversity and community play critical roles in flavor creation. Acetate esters are formed by yeast and bacteria through lipid and acetyl-CoA metabolisms or chemical esterification of alcohols and acids. Bacterial dynamics in relation to organic acid alterations in solid-state AAF were also investigated (Figures 5A). Lactic acid concentrations increased considerably from day one to day 19 in this study. Furthermore, *Lactobacillus*, the most common lactic acid bacteria, was the most common division in AAF. *Lactobacillus* members added a significant amount of lactic acid to vinegar, enhancing a mellow taste by reducing the annoying sour smell. Several yeast strains and molds from the genera *Aspergillus, Penicillium*, and *Candida* were among the citric acid-accumulating microorganisms. Until the end of AAF, the citric acid content gradually increased. Molds and yeast strains were also found to be involved in the AAF process, according to these findings. Furthermore, the concentrations of other organic acids were low, which could be owing to the poor diversity of acetic acid bacteria used in the AAF process (Figure 5A).

The majority of bacteria that produced amino acids were *Corynebacterium glutamicum*, and the increased amino acid concentration could be attributable to yeast autolysis during the vinegar starter and alcohol mash. The *Lactobacillus*, on the other hand, can produce some free amino acids by degrading proteins. The highest levels of free amino acids were found on day 11, followed by a minor decrease on day 17, and an increase on day 19. In the meantime, *Lactobacillus* relative abundance hit new highs. These findings suggested that yeast autolysis may occur in the early and middle stages of AAF, and that the *Lactobacillus* may be involved in the production of free amino acids during the AAF process.

Ester compounds were the main volatiles observed in the vinegar samples. Esters endow fruit-like aroma to vinegar. In this study, *Acetobacter, Norank-c-Cyanobacteria*, and *Weissella* were the three principal contributors to ester production. These microbes are also considered to play key roles in the process of fermentation. Moreover, the microorganisms associated with lipids were the most relevant, and those associated with ketones were the least relevant in this study, and these results are consistent with earlier published studies. On day 4 of AAF, the relative concentration of total esters increased substantially, followed by a drop. On day 4, the relative abundance of the microbial community at the genus level changed dramatically. During AAF, the Acetobacter genus was the most common ethyl acetate-producing bacteria. Many different microorganisms, such as yeast, can improves the production of ethyl acetate. In the first 11 days, the relative level of ethyl acetate increased, followed by a drop, as seen in Table 1. These findings corroborated previous observations that yeasts die out progressively during the AAF process. *Bacillus* sp. can also aid in the formation of esterase and organophosphorus chemicals. *Bacillales* and *Rhodospirillales* can work together to create acetate esters. The synthesis of acetate esters was strongly linked to the succession pattern of relative abundance of the *Bacillus* and *Acetobacter* genera, according to these findings. Furthermore, throughout the AAF process, the relative concentration of carboxylic acids, particularly acetic acid, rose considerably. This enhanced propensity was roughly in line with *Acetobacter* relative abundance dynamics (Figure 5C). These findings suggested that acetic acid was primarily produced from the *Rhodospirillales* family, which was consistent with prior reports indicating fermentation acid generation was primarily due to *Acetobacter* genus metabolism (29). However, starting on day 4, the relative proportion of total alcohol declined considerably, which was reflected in the shift in acetic acid content. The acetic acid bacteria are responsible for the decline of alcohol because ethanol can be converted to acetic acid, whereas yeasts are responsible for the accumulation of alcohol due to the conversion of fermentable carbohydrates into ethanol (Figure 5B). 3-hydroxy-2-butanone (acetoin) is a physiological metabolite secreted by a variety of bacteria that serve as a quality indicator for fermented items and a flavor component in vinegar (30). Acetoin biosynthetic microorganisms in diverse fermentation processes include *Acetobacter, Lactococcus, Klebsiella, Enterobacter,* and *Bacillus*. After day 7 of AAF, the relative concentration of acetoin increased dramatically, which was linked to the high relative abundance of *Lactobacillus* and *Acetobacter* was identified during the AAF process. Furthermore, vinegar is fermented from cereals by numerous bacteria and a variety of taste compounds are created to give vinegar their scent. The principal bioactive components in vinegar have been identified as pyrazine chemicals. Tetramethylpyrazine has piqued interest due to its several bioactivities. It has been widely utilized to treat a variety of ailments, including cardiovascular and hypertension problems.

However, the relative content of tetramethylpyrazine detected in this study was low. Bacillus is a high-yield bacterial strain for tetramethylpyrazine biosynthesis and is closely related to the formation of ligustrazine and its precursors, 2,3-butanedione and 3-hydroxybutanone. The relative content of *Bacillus* in this study was only relatively low (0.34–0.53%), which could be due to the insufficient oxygen flow in the bioreactor.

## 4. Conclusion

The bacterial composition and dynamic succession in the entire solid-state AAF vinegar was studied. *Lactobacillus* and *Acetobacter* are the most common bacteria associated in AAF. Bacterial diversity increased early in the AAF process and subsequently declined afterwards. The bacterial growth during the AAF process was related to pH, titratable acidity, and alcoholic degree. During the entire AAF process, the abundance of lactic acid and acetic acid bacteria was greater than 60%, implying that lactic acid and acetic acid bacteria had a significant impact on vinegar flavor. Furthermore, after AAF, the structure of the bacterial population may be altered to reflects the properties of acidophilic and aerobic communities. The interaction between metabolic processes, bacterial patterns, and fermentation settings are being explored in greater depth, providing new insights into the role of bacterial communities in fermentation. Changes in metabolites during the AAF process were also caused by the dynamics and diversity of microbial population succession. These preliminary findings represent a paradigm shift in our understanding of AAF systems, in which acidophilic and aerobic bacterial communities play critical roles in enhancing alcohol availability and vinegar acetic acid yield. This work established the relationship between bacterial dynamics and metabolite changes in AAF of vinegar production, which might be used as a guide for future AAF fermentation experiments to improve the quality of vinegar.

## Data availability statement

The original contributions presented in the study are publicly available. This data can be found here: NCBI, accession number PRJNA934156.

## Author contributions

WW: conceptualization, data curation, formal analysis, investigation, and writing original draft. FZ: methodology, formal analysis, writing—review and editing, and visualization. XD: writing—review and editing and visualization. YL and JM: investigation, resources, and writing—review and editing. JW: supervision and writing—review and editing. QM and JS: funding acquisition, project administration, supervision, validation, and writing—review and editing. All authors contributed to the article and approved the submitted version.

## Funding

The authors are grateful to the Young Scholar Scientific Research Foundation of Hebei Agricultural University (YJ201850), the Program of the Hebei Youth Top-notch Talent Supporting Plan (0316027), Hebei Province “Three Three Three” Talent Project (A202005002).

## Conflict of interest

The authors declare that the research was conducted in the absence of any commercial or financial relationships that could be construed as a potential conflict of interest.

## Publisher’s note

All claims expressed in this article are solely those of the authors and do not necessarily represent those of their affiliated organizations, or those of the publisher, the editors and the reviewers. Any product that may be evaluated in this article, or claim that may be made by its manufacturer, is not guaranteed or endorsed by the publisher.

## Footnotes

^1^ http://drive5.com/uparse/

^2^ https://sourceforge.net/projects/rdp-classifier/

^3^ https://www.arb-silva.de/
